# cIMP: Synthesis, effector activation, inactivation and occurrence in biological systems

**DOI:** 10.1186/2050-6511-16-S1-A85

**Published:** 2015-09-02

**Authors:** Roland Seifert, Christina Hartwig, Sabine Wolter, Erich Schneider, Heike Bähre, Volkhard Kaever

**Affiliations:** 1 Institute of Pharmacology, Hannover Medical School, Hannover, Germany; 2 Core Unit Metabolomics, Hannover Medical School, Hannover, Germany

## Background

**Figure 1:**
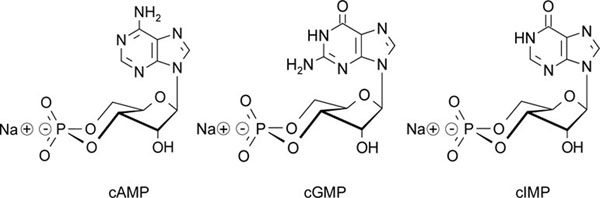
Chemical structures of cAMP, cGMP and cIMP

The soluble nitric oxide (NO)-stimulated guanylyl cyclase (sGC) uses GTP as a substrate to synthesize the secondary messenger cGMP [1]. However, sGC is not so stringent in terms of substrate-specificity and can also use ATP to produce yet another secondary messenger, cAMP [1]. Both cAMP and cGMP induce vasodilation. Recently, it has been proposed that under conditions of hypoxia, sGC predominantly produces cIMP to induce vasoconstriction [2]. Chemically, cGMP and cIMP are very closely related to each other, i.e. the only chemical difference between the two cyclic nucleotides is the missing amino group at the 2’-position of the purine ring (Fig. 1). How can such a small chemical difference between two molecules lead to opposite biological effects? To answer this question, we studied cIMP synthesis, effector activation, inactivation and biological occurrence.

## Methods

We determined cGMP and cIMP concentrations with a highly sensitive and specific HPLC-MS/MS method that was previously described in detail [1,3]. We analysed purified sGCα_1_β_1_ [1], recombinant particulate GC-A (pGC-A) expressed in HEK293 cell membranes or intact HEK293 cells [3], recombinant cAMP- and cGMP-dependent protein kinases (PKA and PKG, respectively) [4], and various recombinant phosphodiesterases (PDEs) [5,6]. We also determined cIMP concentrations in various biological systems [7].

## Results

Purified sGC and recombinant pGC-A expressed in HEK293 cells were very effective at producing cIMP. *K_m_* and *V_max_* values depended on the presence of Mg^2+^ or Mn^2+^ as divalent cation. Under certain conditions, ITP clearly surpassed GTP in terms of *V_max_*. The bacterial toxins CyaA from *Bordetella pertussis* and edema factor from *Bacillus anthracis* also produced cIMP, but efficiency was very low. However, in intact cells we did not obtain any evidence for cIMP production by sGC, pGC-A or bacterial toxins, including ExoY from *Pseudomonas aeruginosa*. Cyclic nucleotides activated PKA isoforms RI and RII in the potency order cAMP > cIMP > cGMP, and PKGI was activated in the potency order cGMP > cIMP ~ cAMP. At PKA, all cyclic nucleotides were full activators, whereas at PKGI , the order of efficacy was cGMP > cIMP > cAMP. With the exception of PDEs 4B and 8A, all other PDEs studied (PDE1A3, 1B, 2A, 3A, 3B, 5A, 6AB, 7A1, 9A and 11A1), hydrolysed cIMP very effectively. In 23 cultured mammalian cell lines from mesenchymal, epithelial and neuronal origin (6 different species), various primary human and rat cells, mouse heart and model organisms including *Caenorhabditis elegans*, *Drosophila melanogaster*, *Saccharomyces cerevisiae*, *Escherichia coli* and *Arabidopsis thaliana* we did not identify cIMP. In contrast, all systems studied contained cAMP, and several systems additionally contained cGMP.

## Conclusions

sGC and pGC-A synthesize cIMP *in vitro*. However, in intact cells, there is no evidence for the occurrence of cIMP and sGC- and pGC-A-mediated cIMP formation. To this end, we have not yet explicitly studied hypoxic conditions. cIMP is a low-potency activator of PKA and PKG. For most PDEs, cIMP is an excellent substrate. This could result in rapid elimination of endogenous cIMP in cells. Our attempts to identify cIMP in organs have been unsuccessful so far. Collectively, presently, we do not have sufficient experimental evidence to support the notion that cIMP serves as second messenger with functions opposite to those of cAMP and cGMP. Extensive studies with biological models under hypoxic conditions are required. In addition, specific cIMP-binding proteins have to be identified. It cannot be excluded that under very specific experimental conditions, cIMP does have a signalling role. One should also keep in mind that cyclic nucleotides can exert biological effects extracellularly via receptors and ion channels.
